# The prognostic study of mental stress-induced myocardial ischemia in coronary revascularization patients with depression/anxiety: rationale and design

**DOI:** 10.1186/s12872-023-03246-3

**Published:** 2023-05-04

**Authors:** Nan Nan, Lei Feng, Wei Dong, Bingyu Gao, Huijuan Zuo, Hongzhi Mi, Gang Wang, Xiantao Song, Hongjia Zhang

**Affiliations:** 1 Department of Cardiology, Beijing Anzhen Hospital, Capital Medical University, No. 2, Anzhen Road, Chaoyang District, Beijing, 100029 China; 2 The National Clinical Research Center for Mental Disorders & Beijing Key Laboratory of Mental Disorders Beijing Anding Hospital, Advanced Innovation Center for Human Brain Protection, Capital Medical University, NO.5 DeWai AnKang Hutong Xicheng District, Beijing, 100088 China; 3 Department of Nuclear Medicine, Beijing Anzhen Hospital, Capital Medical University, No. 2, Anzhen Road, Chaoyang District, Beijing, 100029 China; 4 Department of Community Health Research, Beijing Anzhen Hospital, Capital Medical University, Beijing Institute of Heart Lung and Blood Vessel Disease, No. 2, Anzhen Road, Chaoyang District, Beijing, 100029 China; 5 Department of Cardiovascular Surgery, Beijing Anzhen Hospital, Capital Medical University; Beijing Lab for Cardiovascular Precision Medicine; Key Laboratory of Medical Engineering for Cardiovascular Disease, No. 2, Anzhen Road, Chaoyang District, Beijing, 100029 China

**Keywords:** Mental stress-induced myocardial ischemia, Coronary revascularization, Myocardial perfusion imaging, Endothelial function, Major adverse cardiac events

## Abstract

**Background:**

Mental stress-induced myocardial ischemia (MSIMI) frequently occurs in patients with coronary artery disease (CAD), and is even more common in patients with co-occurring CAD and depression/anxiety. MSIMI appears to be a poor prognostic factor for CAD, but existing data on depression/anxiety patients are limited.

**Methods:**

This cohort study will consecutively screen 2,647 CAD patients between 2023 and 2025. Included subjects will need to have received coronary revascularization and also have depression and/or anxiety at baseline. This study will enroll 360 subjects who meet the criteria. Two mental stress tests will be carried out in each patient at 1 month and 1 year timelines after coronary revascularization, using Stroop color word tests. MSIMI will be assessed by ^99 m−^Tc-sestamibi myocardial perfusion imaging. The endothelial function will be assessed by EndoPAT. Furthermore, we will dynamically monitor patients’ health and mental conditions every 3 months. The mean follow-up time will be 1 year. The primary endpoint is the major adverse cardiac events, a composite of all-cause death, cardiac death, myocardial infarction, stroke, or unplanned revascularization. Secondary endpoints will include overall health and mental conditions. The reproducibility of mental stress combined with myocardial perfusion for detecting MSIMI and comparisons between coronary stenosis and ischemic segments will also be included.

**Conclusions:**

This cohort study will provide information on MSIMI outcomes in CAD patients who also have comorbid depression/anxiety after revascularization. In addition, understanding the long-term dynamics of MSIMI and the match between coronary stenosis and ischemia will provide insight into MSIMI mechanisms.

**Trail Registration:**

ChiCTR2200055792, 2022.1.20, www.medresman.org.cn;

## Background

Mental stress-induced myocardial ischemia (MSIMI) is increasingly recognized as a risk factor and a poor prognostic factor for coronary artery disease (CAD). The relationship between mental stress and myocardial ischemia was originally observed through controlled and objective experiments in 1976 [1]. Over the past 40 years, there has been increasing evidence that mental stress can evoke myocardial ischemia. MSIMI is defined as the imbalance between myocardial oxygen demand and supply during mental stress [2, 3]. MSIMI is common in CAD patients [4–6], and is also a prognostic factor for major adverse cardiovascular events (MACEs). Results from cohort studies have demonstrated that MSIMI is associated with double the risk for subsequent death or adverse cardiovascular events [7–14]. However, the data in high-risk patients (i.e., those with CAD and comorbid depression/anxiety) is limited.

However, no clinical studies on MSIMI have been conducted in patients with comorbid CAD and depression and/or anxiety. Psychological factors (such as depression and anxiety) have interactions with MSIMI in CAD patients. Both depression and anxiety are common psychological factors amongst CAD patients and have emerged as important risk factors for CAD [15]. They also increase the risk of MACEs in CAD patients [15, 16], even beyond the traditional risk factors [17]. Prior work has also found associations between psychological factors and MSIMI in both CAD and myocardial infarction patients [18, 19]. Moreover, the prevalence of MSIMI in patients with depression/anxiety and CAD was 22.08 times higher than in patients without depression/anxiety [20]. Thus, these results may enhance our understanding of the mechanisms underlying the association between MSIMI prognosis and future cardiovascular events in patients with depression and anxiety.

Additionally, long-term dynamic observations of MSIMI are lacking, and due to the overall variability of emotional factors, we speculate that MSIMI may also change over time. A small-sample clinical study using echocardiography to assess MSIMI with decreased ejection fraction found short-term (4 to 8 weeks) repeatability of psychological stress testing, but these findings still need to be replicated in a larger patient population [21].

Therefore, the aim of our study is to observe the dynamical impact of MSIMI on cardiovascular outcomes in a consecutive cohort of CAD patients with depression and/or anxiety who are receiving coronary revascularization and to dynamically observe long-term MSIMI changes. Our main hypothesis is that, amongst CAD patients with comorbid depression/anxiety, those with MSIMI will have higher incidences of MACEs than those without MSIMI.

## Methods

The aim of this study is to dynamically observe the long-term effects of MSIMI on cardiovascular events in high-risk CAD patients combined with depression/anxiety. The mechanism of MSIMI will be further explored, including comparisons between coronary stenosis and myocardial perfusion, endothelial function as well as reproducibility of mental stress.

### Study design and settings

This study is designed as a prospective, single-center, single-blinded cohort study and has been registered at www.medresman.org.cn (trial identifier: ChiCTR2200055792), following the STROBE guidelines [22]. All study processes will be performed at the Beijing Anzhen Hospital. The period of recruitment was from 2023 to 2025. We will have a median follow-up time of 1 year. Data collection will be completed within 1 week of each patients’ completion of the mental stress test. This research has been approved by the Beijing Anzhen Hospital Medical Ethics Committee (NO. 2019001), and all participants provided informed consent.

### Participants

The study participants included adult patients with angiographically-confirmed CAD, including stable or unstable angina pectoris and myocardial infarction. All patients need to have received coronary revascularization procedures, including percutaneous coronary intervention (PCI) or coronary artery bypass grafting (CABG). Before these procedures, we used the Patient Health Questionnaire-9 (PHQ-9) and Generalized Anxiety Disorder-7 (GAD-7) scores to screen for depression and/or anxiety. We used recommended cutoff scores (i.e., PHQ-9 > or = 5 and/or GAD-7 > or = 5) to diagnose the major depressive disorder and generalized anxiety disorder in CAD patients [23]. We also used the Seattle Anginal Questionnaire (SAQ) to assess patients’ health status.

The exclusion criteria were as follows: (1) patients who had acute coronary syndrome within 1 week; (2) patients with severe heart failure (NYHA ≥ III); (3) patients with uncontrolled hypertension (≥ 180/110mmHg); (4) patients with severe mental illness, except for severe depression; (5) patients with contraindications to nuclear medicine, such as active asthma; (6) dialysis patients; (7) patients with life expectancies less than 1 year, such as those with malignant tumor; (8) patients who were blind or were unable to read a single word of the 4 colors on the Stroop test. The complete inclusion and exclusion criteria are shown in Table 1.

**Table 1:** Study Inclusion and Exclusion Criteria

Study Inclusion Criteria	Study Exclusion Criteria
1. Age ≥ 18 years	1. Acute coronary syndrome within 1 week
2. Angiographically- confirmed CAD	2. Severe heart failure (NYHA ≥ III)
3. Received coronary revascularization for 1 month	3. Uncontrolled hypertension (≥ 180/110 mmHg)
4. PHQ-9 > or = 5 and/or GAD-7 > or = 5	4. Severe mental illness (except for severe depression)
	5. Contraindications to nuclear medicine such as active asthma
	6. Dialysis patients
	7. Life expectancy less than 1 year
	8. Blind or illiterate patients

### Measures

#### Mental stress

Each participant will take two mental stress tests at 1 month and 1 year times points after coronary revascularization. The mental stress protocol includes the Stroop color word test (SCWT), which conflicts with the color and meaning. The SCWT is well-accepted and has good reproducibility amongst CAD patients [24]. The SCWT process has been detailed in previously published articles [25]. Both blood pressure (BP) and heart rate (HR) will be measured before SCWT and every 1 min after the test begins. After the test is completed, each patient will have 5 min to rest. Myocardial oxygen demand will be estimated by the rate-pressure product (RPP), which is defined as the peak HR multiplied by the maximum BP during the test. The test requires two trained operators to perform and record.

### SPECT imaging procedures

**Fig. 1:**
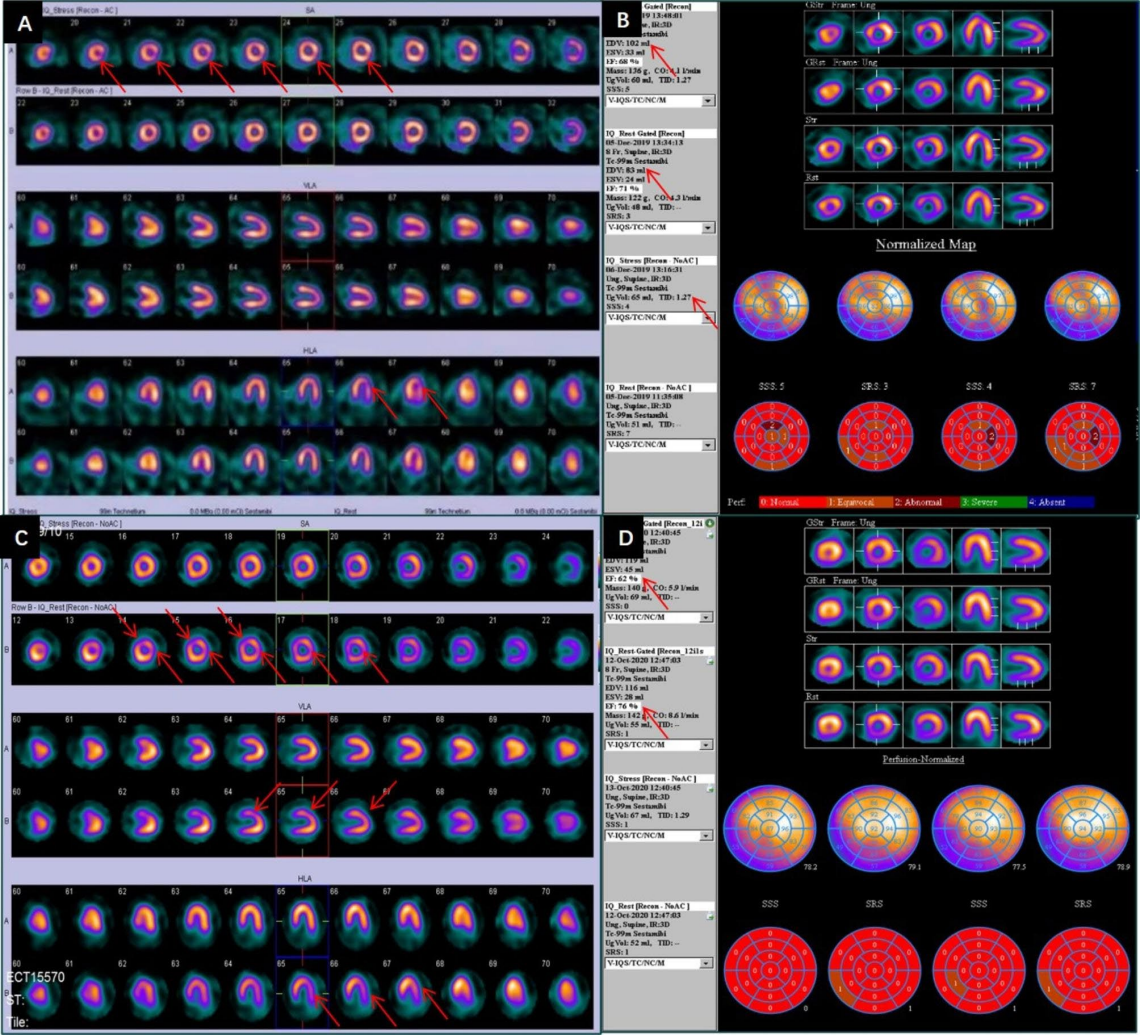
Four abnormal SPECT phenomena (**A**) RMPD: The stress SPECT images show a reversible perfusion defect from the lateral wall apex to part of the middle of the posterior lateral wall. (**B**) TID: Rest EDV is 83ml and stress EDV is 102ml. The TID value is 1.27, which is likely to represent diffuse subendocardial ischemia or microvascular disease in the absence of epicardial coronary disease. (**C**) RR: The stress images show no perfusion defects, while the rest images show perfusion defects (including in the apical anterior segment of the left ventricle wall, the lateral apical segment, the posterior lateral wall mid-segment, and the basal segment). (**D**) EF decline ≥ 5%: Rest EF was 76%, and stress EF is declined to 62%; EF declined 14%

MSIMI will be evaluated using ^99 m−^Tc-sestamibi gated Single Photon Emission Computed Tomography (SPECT) scans. The scanning and post-processing processes have been explained in detail in previous articles [25]. Tridimensional reconstruction of the left ventricle will be performed to assess ventricular function, in addition to analyzing ejection fraction (EF), end-diastolic volume (EDV), end-systolic volume (ESV), contractility, and myocardial thickness [24]. Four abnormal SPECT phenomena will be considered positive for MISIMI [26, 27], including reversable myocardial perfusion defects (RMPD), transient ischemic dilation (TID), reverse redistribution (RR), and EF reduction of ≥ 5% [28] (see Fig. 1). SPECT images will be analyzed by two experts who did not know the clinical conditions, and the controversial parts need to consult with a third expert to determine.

### Comparison between coronary angiography and SPECT imaging

The mechanism underlying MSIMI is not clear, but microvascular dysfunction maybe one underlying factor [5]. However, a series of MSIMI studies found that the SPECT images of MSIMI and conventional stress-induced myocardial ischemia (CSIMI) did not match [29–31], but there were no studies that matched 17-segment MSIMI models and coronary angiographic vascular stenosis. Because all patients in this study received psychological stress SPECTs and coronary angiography, we can use their data to compare images to find the imaging-based characteristics of MSIMI.

### Health and mental health conditions

CAD-related health conditions will be evaluated using the SAQ Questionnaire, which is one of the most widely used and frequently studied health status end points that exists within cardiovascular medicine [32]. Higher scores indicate better health status [33]. Mental status, which mainly included depression and anxiety in our study, will be assessed with the PHQ-9 and GAD-7 questionnaires at baseline and at each follow-up time point. The accuracy of the PHQ-9 and GAD-7 for screening depression and anxiety in CAD patients has been demonstrated across several previous studies [34–36]. Following previously-published suggestions [25], we defined the presence of depression or anxiety by a score of 5 points or higher. We will assess CAD-related health and mental status at baseline and at every follow-up time point after coronary revascularization.

### Endothelial function test

Non-invasive detection of peripheral endothelial function tests will be used to assess the relationship between endothelial dysfunction and MSIMI. Endothelial function tests will be implemented before mental stress tests at baseline and follow-up time points. An EndoPAT 2000 Machine (Itamar Medical Ltd, Caesarea, Israel) [37] will be used to assess the reactive hyperemia index (RHI). Nitrates or any medications for erectile dysfunction will be discontinued 1 day before each patient’s endothelial function test. The test is performed by a trained physician.

### Data collection

Data will be collected by a pre-designed Case Report Form. Data elements will include patients’ demographics, medical histories, SAQ questionnaires, depression and anxiety assessments, medical care records, medications, laboratory results, coronary angiography results, mental stress tests, SPECT imaging results, endothelial function tests, follow-up questionnaires, and clinical event documentations (Table 2).

**Table 2:** Data collection DM, diabetes mellitus; CKD: coronary kidney disease; PCI: percutaneous coronary intervention; CABG: coronary artery bypass grafting; MI: myocardial infarction; PHQ-9: Patient Health Questionnaire-9; GAD-7: Generalized Anxiety Disorder-7; SAQ: Seattle Angina Questionnaire; SBP: systolic blood pressure; DBP: diastolic blood pressure; HR: heart rate; ECG: electrocardiograph; BMI: body mass index; CCB: calcium channel blocker; ACEI/ARB: angiotensin-converting enzyme inhibitor/angiotensin receptor blocker; BNP: brain natriuretic peptide; CRP: C-reactive protein; LDL-C: low-density lipoprotein cholesterol; LVEF: left ventricular ejection fraction; UCG: ultrasonic cardiogram; CAG: coronary angiography; LM: left main; SYNTAX: Synergy between PCI with Taxus and Cardiac Surgery; RPP: Rate-Pressure Product; TPD: total perfusion deficit; EDV: end-diastolic volume; ESV: end-systolic volume; EF: ejection fraction; RHI: reactive hyperemia index;

Category	Data elements
**Patients’ demographics**	**Age, sex, ethnicity, current address, phone number, education, occupation, and marriage status**
**Medical histories**	**History of hypertension, dyslipidemia, DM, peripheral vascular disease, cerebrovascular disease, tumor, CKD, post PCI/CABG, post MI, mental disease; cigarette smoking; drinking and family history**
**SAQ questionnaire**	**19-item SAQ questionnaire, including angina frequency, physical limitations, angina stability, treatment satisfaction, and quality of life**
**Depression and anxiety assessment**	**PHQ-9 and GAD-7**
**Admission**	**Admission time; symptoms, admission SBP/DBP, weight, height, HR; diagnostic; cardiac function classification; ECG findings; BMI**
**Medications**	**Aspirin; clopidogrel; ticagrelor; statins; ezetimibe; other lipid-lowering drugs; β-blockers; CCB; ACEI/ARB; nitrates; diuretics; hypoglycemic agents**
**Laboratory results**	**Routine blood tests; platelet function tests; glucose; HBA1c; creatinine; troponin; CK-MB; BNP; hsCRP; LDL-C; LVEF assessment by UCG**
**Coronary angiography**	**CAG time; PCI/CABG; number of vascular lesions; LM lesion; SYNTAX score**
**Mental stress test**	**SBP, DBP, HR and RPP at rest and 1-5 min during mental stress**
**SPECT imaging results**	**TPD, EDV, ESV, and EF at rest and mental stress.**
**Endothelial function test**	**RHI**
**Follow-up questionnaires**	**SAQ, PHQ-9 and GAD-7 questionnaire; medications;**
**Clinical events**	**All-cause death, cardiac death, any MI, stroke, or any unplanned revascularization**

### Follow-Up

Patients will be followed up every 3 months after coronary revascularization with telephone calls or clinical visits for a mean follow-up of 1 year. SAQ, PHQ-9, and GAD-7 questionnaires will be used to assess health and mental status every 3 months. At 1 month and 1 year, patients will need to have a clinical visit to receive mental stress tests, SPECT imaging, and assessments of psychological and health status. At 1 year, patients will receive coronary angiography. At the 3, 6 and 9-month follow-up time points, patients will be contacted by phone call to assess psychological and health status, as well as study-related events. All patients will receive optimal medical therapy according to clinical practice guidelines.

### Endpoints

**Fig. 2:**
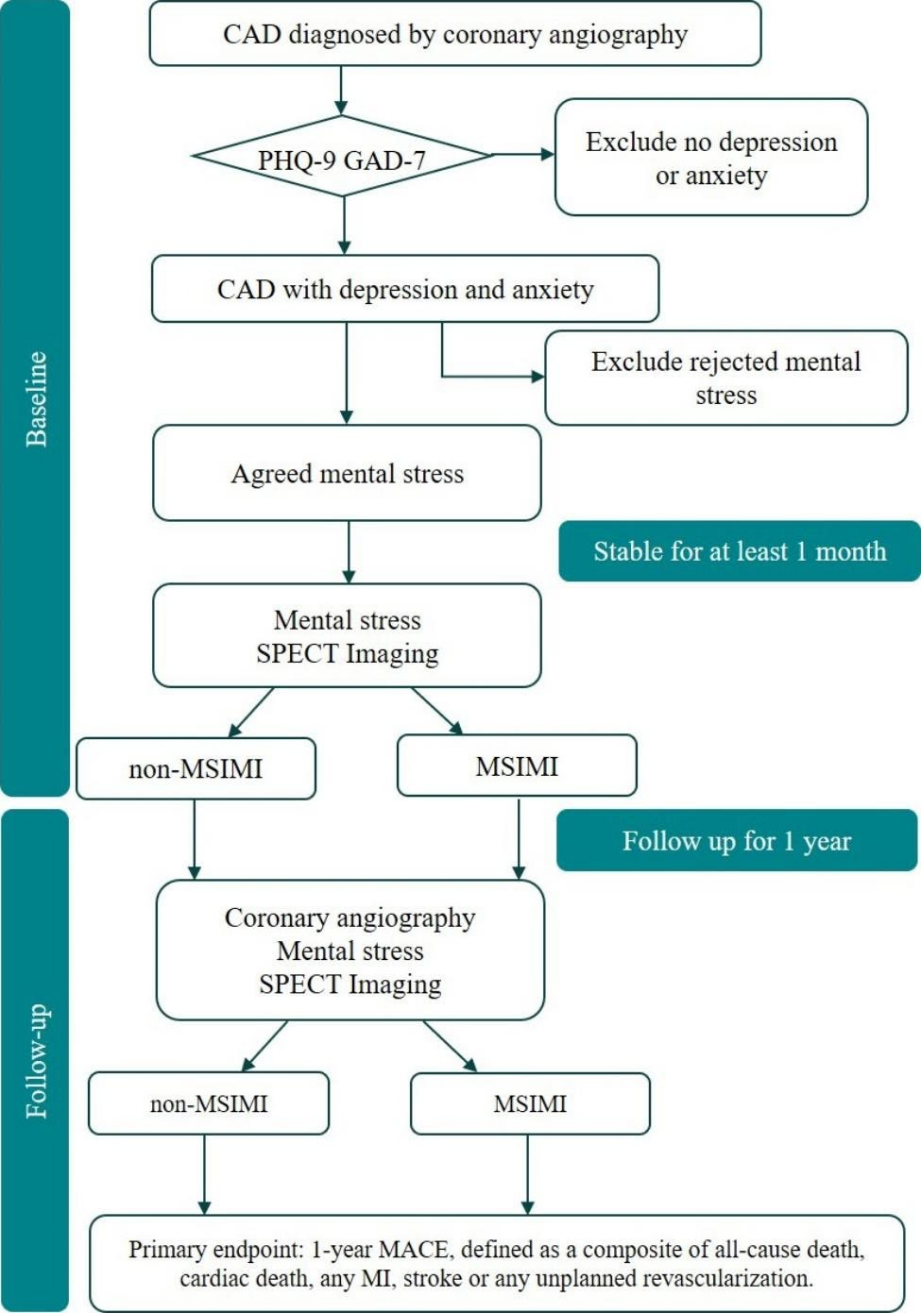
Flowchart of the MSIMI Cohort Study in CAD Patients with comorbid Depression/anxiety after Coronary Revascularization

The primary will be the one-year difference rate of MACEs between patients with and without MSIMI. MACEs are defined as a composite of all-cause death, cardiac death, non-fatal MI, non-fatal stroke or any unplanned revascularization. Secondary endpoints will include health and mental status as well as changes in endothelial function in MSIMI. The reproducibility of mental stress combined with myocardial perfusion for detecting MSIMI will also be evaluated in this study. The study flowchart is displayed in Fig. 2.

### Statistics

The hypothesis of our prospective cohort study is that MSIMI will increase the rate of 1-year MACEs after coronary revascularization in patients with comorbid CAD and depression and/or anxiety. Sample size calculation is based on the primary endpoint. According to a previous systematic review and meta-analysis [7], we estimated that the presence of MSIMI would result in a MACEs rate at 1 year after coronary revascularization that is 2.24 times higher than patients without MSIMI (33.82% vs. 15.37%). According to our previous meta-analysis, compared with non-depressed people, the rate of MACEs in depressed patients is between 8.0 and 44.2%, the average rate is 28.7% [38]. Assuming a loss to follow-up rate of 5%, we calculated that 360 CAD patients after revascularization combined with derpression/anxiety would need to be enrolled in order to detect this expected difference with 85% power at the 5% significance level. According to our center’s statistics, the prevalence of depression and/or anxiety was 13.6% [39], these 360 CAD patients with depression/anxiety after revascularization need to be screened from 2,647 CAD patients. These findings were calculated with PASS 11.0.7 statistical software (NCSS, USA).

Continuous variables will be expressed as mean ± SD or median with corresponding IQR, and differences in continuous data will be compared by using Students t-tests or Mann-Whitney U tests as appropriate. Categorical variables will be expressed as frequency counts and will be compared by using chi-square tests or Fishers exact tests. The association between MSIMI and clinical measurements will be assessed with univariable and multivariable logistic regression. Kendall’s tau-b correlation coefficient test will be used to detect the consistency of MSIMI at baseline and follow-up time points. The impact of MSIMI alone at 1 month and 12 month after revascularization on MACEs at 12-month follow-up was compared by Kaplan–Meier curve survival analysis. To identify predictors, the influence of combined MSIMI, mental status and endothelial function on MACEs will be analysed by Cox proportional hazards regression models. A value of P < 0.05 was considered statistically significant. Statistical analyses will be performed using IBM SPSS Statistics 22.0.

## Discussion

Our cohort study will be the first clinical trial to attempt to explore the prognosis of MSIMI in high-risk CAD patients with comorbid depression and/or anxiety following coronary revascularization. The major strengths and features of our study include: (1) a large sample size of consecutive CAD patients with depression and/or anxiety; (2) the ability to verify the reproducibility of MSIMI and its long-term impacts on MACEs; and (3) the ability to examine comprehensive myocardial perfusion and coronary angiography during times of psychological stress.

The American Heart Association recommends that all CAD patients be screened for depression because of its incidence and association with poor prognosis [39]. Additionally, standardized screening pathways for early identification of depression have been shown to improve outcomes [40]. Anxiety is also common in CAD and is confirmed to be associated with MACE events, but the relationship is not as strong as the relationship with depression [15, 16, 41]. Additionally, compared to CAD patients without depression or anxiety, the prevalence of MSIMI has been shown to be 22.08 times higher in CAD patients with depression and/or anxiety [20]. However, the characteristics of MSIMI in CAD with depression/anxiety and the prognostic outcomes are still not well-understood. Thus, we will screen CAD patients for depression and anxiety based on AHA recommendations to determine the characteristics and prognosis of these high-risk patients via long-term follow-up of mental status, MSIMI, and MACE events.

While previous MSIMI cohort studies on prognosis only performed only one mental stress test at baseline [7, 13], our study will dynamically observe changes in MSIMI with two mental stress tests, both at baseline and follow-up. At the same time, according to the results of two psychological stress tests, the patients were divided into four groups: MSIMI negative group, MSIMI positive group, MSIMI trans-negative group and MSIMI trans-positive group, and the differences in the occurrence of MACE events among the four groups were observed.

Few previous studies have investigated MSIMI reproducibility. Most studies have included only one mental stress test [4, 6, 7, 11, 13, 14]. In a recent study [42], 12 of 16 (75%) CAD patients showed repeated MSIMI by radionuclide ventriculography at an approximately 2-week interval. Additionally, the 3 CAD patients who did not exhibit mental stress-induced ischemia also did not exhibit ischemia with repeated mental stress testing. The PIMI study [43] compared the two standardized mental stress tests (a timed SCWT and a public speaking task) in a nuclear cardiology laboratory before and after 2–8 weeks. 68% and 60% of the patients had consistent scores on the Stroop test and speech test, respectively, which was considered modestly reproducible. However, neither study lasted longer than 2 months. The results of our study will examine long-term reproducibility over the course of 12 months and will link the findings with MACE events.

Most previous studies have shown no correlation between coronary angiographic stenosis and the occurrence of MSIMI [31, 44], and it has been speculated that MSIMI is driven by alternative mechanisms, such as endothelial and microvascular dysfunction [5]. However, these studies did not simultaneously perform coronary angiography and SPECT imaging after mental stress, meaning it was impossible to understand the relationship between coronary stenosis and MSIMI segments. Therefore, our study design will include patients with coronary angiography and revascularization, and will thus be able to compare whether the blood vessels corresponding to ischemic MSIMI segments are related to stenosis or revascularization.

One limitation of our study is that we did not assess CSIMI, so we could not simultaneously obtain coronary flow reserve (CFR) data or perform adenosine stress testing. In the pre-experimental phase, we tried to measure CFR during mental stress tests in 5 patients. However, all patients had CFR values less than 2.0, so we considered mental stress to be less intense than adenosine. Thus, we were unable to assess microvascular dysfunction based on CFR in this population. We will consider correcting this oversight in future research.

## Conclusion

Our MSIMI study is a prospective, single-center, single-blinded cohort study that will assess MSIMI in CAD patients with comorbid depression and/or anxiety after revascularization. Our hypothesis is that MSIMI will enhance the rate of 1-year MACE events. The study will also provide evidence for the long-term dynamics of mental stress with ^99 m−^Tc-sestamibi SPECT imaging, as well as the interpretation of imaging mechanisms by contrasting coronary angiography with stenotic coronary arteries and SPECT ischemic segments.

## Data Availability

Not applicable.

## List of abbreviations

MSIMI: Mental stress-induced myocardial ischemia
CAD: Coronary artery disease
MACEs: Major adverse cardiovascular events
PCI: Percutaneous coronary intervention
CABG: Coronary artery bypass grafting
PHQ-9: Patient Health Questionnaire-9
GAD-7: Generalized Anxiety Disorder-7
SAQ: Seattle Anginal Questionnaire
SCWT: Stroop color word test
BP: Blood Pressure
HR: Heart rate
RPP: rate-pressure product
SPECT: Single Photon Emission Computed Tomography
EF: Ejection fraction
EDV: End-diastolic volume
ESV: End-systolic volume
RMPD: Reversable myocardial perfusion defects
TID: Transient ischemic dilation
RR: Reverse redistribution
CSIMI: Conventional stress-induced myocardial ischemia
RHI: Reactive hyperemia index
CFR: Coronary flow reserve
